# Cost-Effectiveness Evaluation of Add-on Empagliflozin in Patients With Heart Failure and a Reduced Ejection Fraction From the Healthcare System's Perspective in the Asia-Pacific Region

**DOI:** 10.3389/fcvm.2021.750381

**Published:** 2021-10-29

**Authors:** Chia-Te Liao, Chun-Ting Yang, Fang-Hsiu Kuo, Mei-Chuan Lee, Wei-Ting Chang, Hsin-Ju Tang, Yi-Ming Hua, Hung-Yu Chang, Zhih-Cherng Chen, Carol Strong, Huang-Tz Ou, Han Siong Toh

**Affiliations:** ^1^Department of Public Health, College of Medicine, National Cheng Kung University, Tainan, Taiwan; ^2^Division of Cardiology, Department of Internal Medicine, Chi Mei Medical Center, Tainan, Taiwan; ^3^Department of Electrical Engineer, Southern Taiwan University of Science and Technology, Tainan, Taiwan; ^4^Institute of Clinical Pharmacy and Pharmaceutical Sciences, College of Medicine, National Cheng Kung University, Tainan, Taiwan; ^5^Department of Pharmacy, Chi Mei Medical Center, Tainan, Taiwan; ^6^Institute of Clinical Medicine, College of Medicine, National Cheng Kung University, Tainan, Taiwan; ^7^Department of Biotechnology, Southern Taiwan University of Science and Technology, Tainan, Taiwan; ^8^Department of Nursing, Chang Gung University of Science and Technology, Chiayi, Taiwan; ^9^Faculty of Medicine, School of Medicine, National Yang Ming Chiao Tung University, Taipei, Taiwan; ^10^Heart Center, Cheng Hsin General Hospital, Taipei, Taiwan; ^11^Department of Pharmacy, College of Medicine, National Cheng Kung University, Tainan, Taiwan; ^12^Department of Intensive Care Medicine, Chi Mei Medical Center, Tainan, Taiwan; ^13^Department of Health and Nutrition, Chia Nan University of Pharmacy & Science, Tainan, Taiwan

**Keywords:** cost-effectiveness, empagliflozin, SGLT2 inhibitor, heart failure with a reduced ejection fraction (HFrEF), systolic heart failure, Asia-Pacific

## Abstract

**Background:** EMPEROR-Reduced trial provides promising evidence on the efficacy of empagliflozin adding to the standard treatment in patients with heart failure and reduced ejection fraction (HFrEF). This study aimed to investigate the cost-effectiveness of add-on empagliflozin vs. standard therapy alone in HFrEF from the perspective of the Asia-Pacific healthcare systems.

**Methods:** A Markov model was constructed to simulate HFrEF patients and to project the lifetime direct medical costs and quality-adjusted life years (QALY) of both therapies. Transitional probabilities were derived from the EMPEROR-Reduced trial. Country-specific costs and utilities were extracted from published resources. Incremental cost-effectiveness ratio (ICER) against willingness to pay (WTP) threshold was used to examine the cost-effectiveness. A series of sensitivity analyses was performed to ensure the robustness of the results.

**Results:** The ICERs of add-on empagliflozin vs. standard therapy alone in HFrEF were US$20,508, US$24,046, US$8,846, US$53,791, US$21,543, and US$20,982 per QALY gained in Taiwan, Japan, South Korea, Singapore, Thailand, and Australia, respectively. Across these countries, the probabilities of being cost-effective for using add-on empagliflozin under the WTP threshold of 3-times country-specific gross domestic product per capita were 93.7% in Taiwan, 95.6% in Japan, 96.3% in South Korea, 94.2% Singapore, 51.9% in Thailand, and 95.9% in Australia. The probabilities were reduced when shortening the time horizon, assuming the same cardiovascular mortality for both treatments, and setting lower WTP thresholds.

**Conclusion:** Adding empagliflozin to HFrEF treatment is expected to be a cost-effective option among the Asia-Pacific countries. The cost-effectiveness is influenced by the WTP thresholds of different countries.

## Background

Heart failure (HF) is a clinical syndrome manifesting the final status of most cardiovascular diseases (1). Globally, an estimated HF prevalence is between 1 and 2% of the adult population, and the prevalence is estimated to be 1.3–6.7% in East Asia (2, 3). During the last few decades, the prevalence continues to grow with the rapidly aging population and improving healthcare for critical cardiovascular diseases (4, 5). Particularly, the prevalence soars up to more than 10% in populations aged 70 years or older in developed countries (6). Thus, the worldwide financial burden of HF care is projected to increase substantially in the following decades (7).

At present, several sodium-glucose cotransporter 2 (SGLT2) inhibitors, which were initially developed as glucose-lowering agents for type 2 diabetes, have been shown promising benefits to reduce the risk of hospitalization for HF (HHF) and cardiovascular death regardless of the presence or absence of diabetes (8, 9). The Cardiovascular and Renal Outcomes with Empagliflozin in Heart Failure (EMPEROR-Reduced) trial is a large-scale, multinational, multicenter, double-blind, randomized controlled trial to investigate the effects of SLGT2 inhibitors on cardiovascular outcomes among patients with heart failure and a reduced ejection fraction (HFrEF). In 3,730 HFrEF patients with or without type 2 diabetes mellitus, the addition of empagliflozin (10 mg once daily) reduces cardiovascular mortality by 31% and hospitalization for progression of HF by 8%, when compared to the standard guideline-directed medical therapy alone (9).

However, in addition to clinical effectiveness, the economic benefits also play an essential role in healthcare decision making. Although another SGLT2 inhibitor, dapagliflozin, has been shown to be a cost-effective add-on therapy for HFrEF in some countries (10–12), the cost-effectiveness data of empagliflozin in HFrEF treatment remains sparse. Besides, cost-effectiveness may be altered due to the diversity of healthcare systems across different countries. Until now, health economic evaluation of adding empagliflozin to standard care for HFrEF populations in the Asia-Pacific countries remains lacking. Thus, to fill this gap, the objective of this study is to assess the cost-effectiveness of add-on empagliflozin to standard therapy vs. standard therapy alone in HFrEF patients from a healthcare system's perspective in Taiwan and other Asia-Pacific countries.

## Methods

### Rationale and Structure of Model

**Figure 1:**
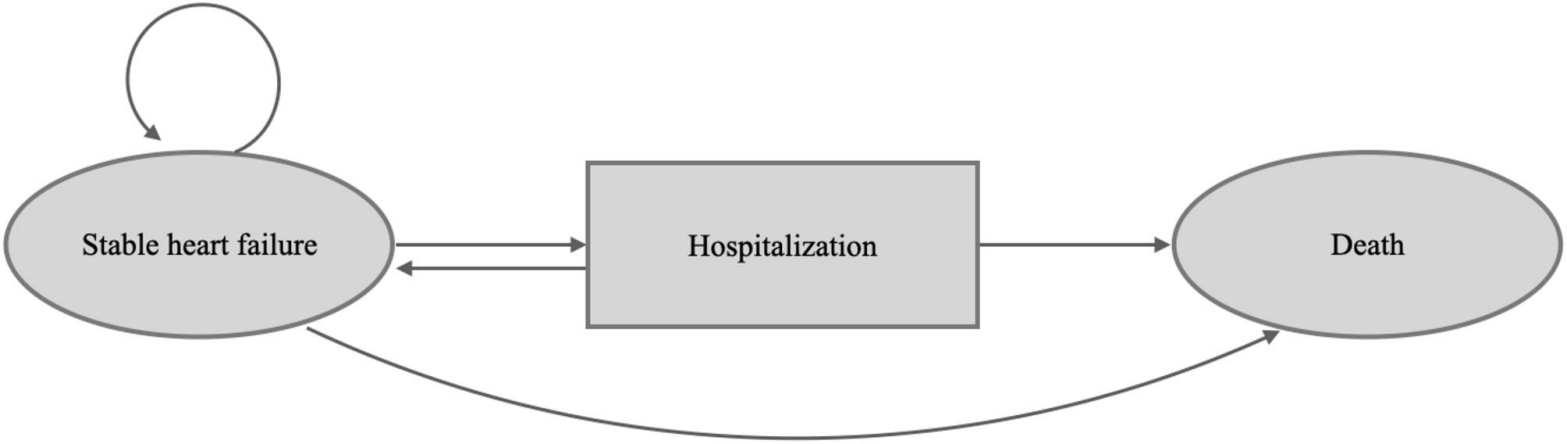
Patients occupy health states, shown in the ovals. Patients transition from different health states represented as arrows based on transition probabilities.

This study constructed a decision model and Markov model to assess the pharmacoeconomic benefit of empagliflozin (10 mg once daily) added to standard therapy vs. standard therapy alone in patients with HFrEF in Taiwan and other Asia-Pacific countries. The model followed the standard structure of the HF model (13, 14), wherein each month, all patients with HF have a risk of either stable HF without further adverse effects, hospitalization for acute decompensated HF, or death (Figure 1, Supplementary Figure 1). This two-state Markov model simulated HF patients for the Taiwanese and other Asia-Pacific populations, and data on efficacy and safety were adopted from the EMPEROR-Reduced trial (9). We simulated subjects with equivalent characteristics as the trial population. Additionally, we modeled the costs and health utilities for the time horizon of 15 years.

The decision analysis estimated the economic outcomes, including lifetime medical costs, life years, quality-adjusted life years (QALYs), and incremental cost-effectiveness ratio (ICER) (15). The model used 1 month as each cycle duration because the acute stage usually lasts 1 month. In the base-case analysis, the starting age for the simulated subjects was 67 years old according to the EMPEROR-Reduced trial, and all subjects progressed from stable HFrEF without acute events through the Markov model until death or until a 15-year horizon, which is close to the life expectancy of the Asia-Pacific countries (16, 17). Cost and utility data were discounted by an annual rate of 3% according to the Taiwan Guidelines of Methodological Standards for Pharmacoeconomic Evaluation. All the analyses were conducted on Microsoft Excel, SAS software V9.4 and TreeAge 2020. This study was granted exemption from review by the Ethics Committee of Chi Mei Medical Center (Ref.11005-E02).

### Model Population, Model Assumption, and Transitional Probability

The study modeled a population that was similar to the EMPEROR-Reduced trial. Briefly, the eligibility criteria of patients enrolled in the trial included age ≥ 18 years, left ventricular ejection fraction of ≤ 40%, New York Heart Association functional class (NYHA Fc) II-IV and N-terminal pro-brain natriuretic peptide > 600 pg/mL (1,200 pg/mL for patients with atrial fibrillation). All patients were required to receive standard HF care, including diuretics, inhibitors of the renin-angiotensin system and neprilysin, beta-blockers, mineralocorticoid receptor antagonists, and, when indicated, cardiac devices. The detailed inclusion and exclusion criteria of the EMPEROR-Reduced trial and the baseline characteristics of the patients have been presented elsewhere (9). The patients were followed up for a median of 16 months, and the primary endpoints were hospitalization due to worsening heart failure or death from cardiovascular causes.

In this model, the populations using standard therapy with or without empagliflozin (10 mg once daily) were assumed to have stable HF status at the beginning, with no clinical events occurring in the first cycle. Then, the modeled patients moved onto the next status, i.e., hospitalization or death, based on the corresponding transitional probability. Monthly transitional probabilities were converted from annual transitional probabilities, which were derived from the proportion of given events occurring over a median follow-up period of 16 months from the EMPEROR-Reduced trial (9, 18) (Table 1, Supplementary Table 1).

**Table 1:** Input parameters for base-case analysis in the model in Taiwan setting.

**Variables**	**Estimates**	**Standard error/Range**	**Distribution**	**References**
**Transitional probabilities**
**Hospitalization for heart failure**			Beta	EMPEROR-Reduced trial (9)
Add-on empagliflozin	0.008811915	0.002165244		
Standard therapy alone	0.012566527	0.002578038		
**Cardiovascular death**				
Add-on empagliflozin	0.006589325	0.00187447		
Standard therapy alone	0.007131185	0.001947398		
**All-cause death**				
Add-on empagliflozin	0.002113141	0.001063894		
Standard therapy alone	0.002177683	0.001078827		
**Utility score**
Stable heart failure	0.770	0.016	Beta	(10, 19)
Decrement for age	−0.0016	0.0001		
Hospital for heart failure	−0.321	0.02		
**Monthly costs (US$)**
Monthly costs of empagliflozin (10 mg once per day)	35	17.5	Gamma	NHIRD
Monthly costs of stable heart failure	450	225		
Mean costs of hospitalization for heart failure	2,887	1,443.5		
Costs before cardiovascular death	3,430	1,715		
Costs before all-cause death	3,390	1,695		

*NHIRD, National Health Insurance Research Database*.

*The monthly transition probabilities were transformed by the following process*.

*(1) Probability (obtained from the EMPEROR-Reduced trial) to a rate = [-ln (1-p)] ÷ t*.

*(2) Rate to a probability (monthly transition probability applied in the analyses) = 1- exp(-rt)*.

*Where r = rate, p = probability, and t = time*.

*Example: the probability of hospitalization for heart failure is 13.2% over the follow-up period in the EMEPROR-Reduced trial*.

*16-month probability was transformed to one-month rate and then one-month rate was transformed to one-month probability*.

*one-month rate = (-ln (1-0.132)/16 = 0.00885*.

*one-month possibility = 1-exp (−0.00885) = 0.008811915*.

Patients in the model who underwent hospitalization due to HF decompensation either moved back to stable HF status or death after the acute stage. The different statuses were assumed to be independent without interaction and were not allowed to occur simultaneously. The transitional probability of each status was constant over time. One-off treatment costs were only obtained for acute stages of HHF and cardiovascular death. Furthermore, the treatment effect was assumed to be consistent throughout.

### Utilities and Costs

Utility scores were applied for each cycle for patients in the HF state based on EQ-5D scores or the Kansas City Cardiomyopathy Questionnaire (KCCQ) scores obtained from the published studies (10, 19). Utilities were assumed to be the same across both treatment regimens. Since HHF and aging are not chronic conditions, disutility was used in the model (19, 20). Input annual utility scores are presented in Table 1.

Costs for HF management were estimated among populations with chronic HF, identified from Taiwan's National Health Insurance Research Database (NHIRD). People who met both the following criteria were defined as patients with chronic HF: (1) ≥2 diagnoses of HF in the outpatient care department within 180 days in 2015, and (2) without any admission or emergency visits for HF in the previous 180 days of the first HF diagnosis in 2015.

To measure the cost of chronic HF care, chronic HF patients were followed up until the development of an acute HF event (i.e., hospitalization or emergency visit for HF), death, or the end of December 2018, whichever came first. Chronic HF costs were calculated as the sum of medical expenditure divided by the total number of followed person-months. For the cost estimation of HF hospitalization, patients with HF events were identified first, and the medical costs in the first and the following months were measured separately as model inputs. Regarding death, death cases and death causes were identified through the Cause of Death files in the NHIRD, and the medical costs within 1 month before cardiovascular and non-cardiovascular death were estimated. In the analysis, the cost was updated for inflation to 2020 using the medical consumer price index and are presented in US dollars (US$).

### Base-Case Cost-effectiveness Analysis

The model was run with a time horizon of 15 years (180 cycles). We projected the discounted lifetime healthcare costs by multiplying the number of subjects with the sum of the costs in every health status. QALY was estimated using the utility values associated with each health status multiplied by the proportion of subjects living in that status. Total QALYs and life years were accumulated from the QALY and life year values in each cycle. ICER, including costs per QALY and life year gained, was calculated by dividing the incremental costs by the incremental QALYs and life years. We applied the willingness to pay (WTP) threshold of US$25,000 and 75,000, which was close to the one-time (1x) and three-times (3x) gross domestic product (GDP) per capita of Taiwan in 2020, to determine if add-on empagliflozin vs. standard care alone in HFrEF was a very cost-effective (i.e., ICER ≤ US$25,000) or only a cost-effective (i.e., ICER ≤ US$75,000) option (21, 22).

### One-Way Sensitivity Analyses

**Figure 2:**
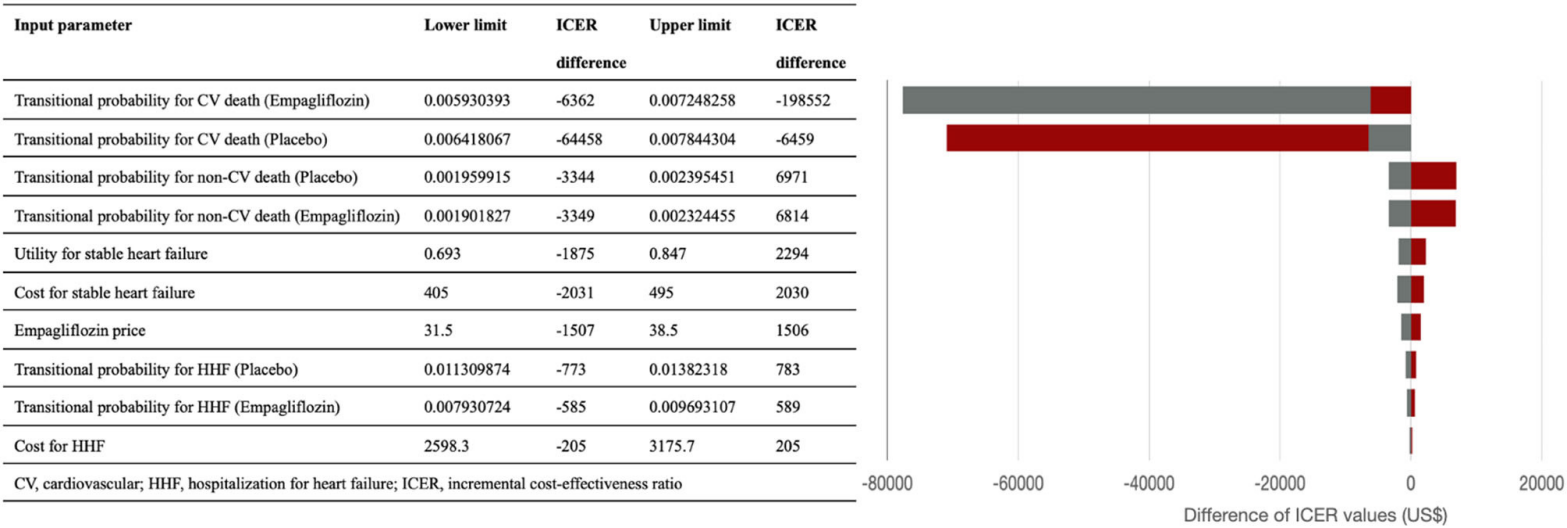
The tornado diagram presents the results of one-way sensitivity analysis. The red and gray bar represent the corresponding incremental cost-effective ratio values when the lower and upper limits of the parameters were used. ICER, incremental cost-effectiveness ratio; CV, cardiovascular; HHF, Hospitalization for heart failure.

We performed one-way sensitivity analysis with varying values for all input parameters through plausible ranges (± 10%) or alternative values to evaluate the robustness of our cost-effectiveness analysis results. The results are presented as a tornado diagram in Figure 2.

### Probabilistic Sensitivity Analyses

To assess the intra-individual and parameter uncertainties, we conducted a probabilistic sensitivity analysis (PSA) by the Monte Carlo Simulation (23), in which subjects were randomly sampled and simulations were repeated 1,000 times to obtain the outcomes. As for the input variable ranges in the simulation, beta distribution was used for transitional probabilities, beta distribution for utilities (utility value ranged between 0 and 1), and gamma distribution for costs (costs could not be <0) (24). The PSA results are presented in the cost-effectiveness acceptability curve (Supplementary Figures 3, 4).

### Scenario Analyses in the Asia-Pacific Countries

**Figure 3:**
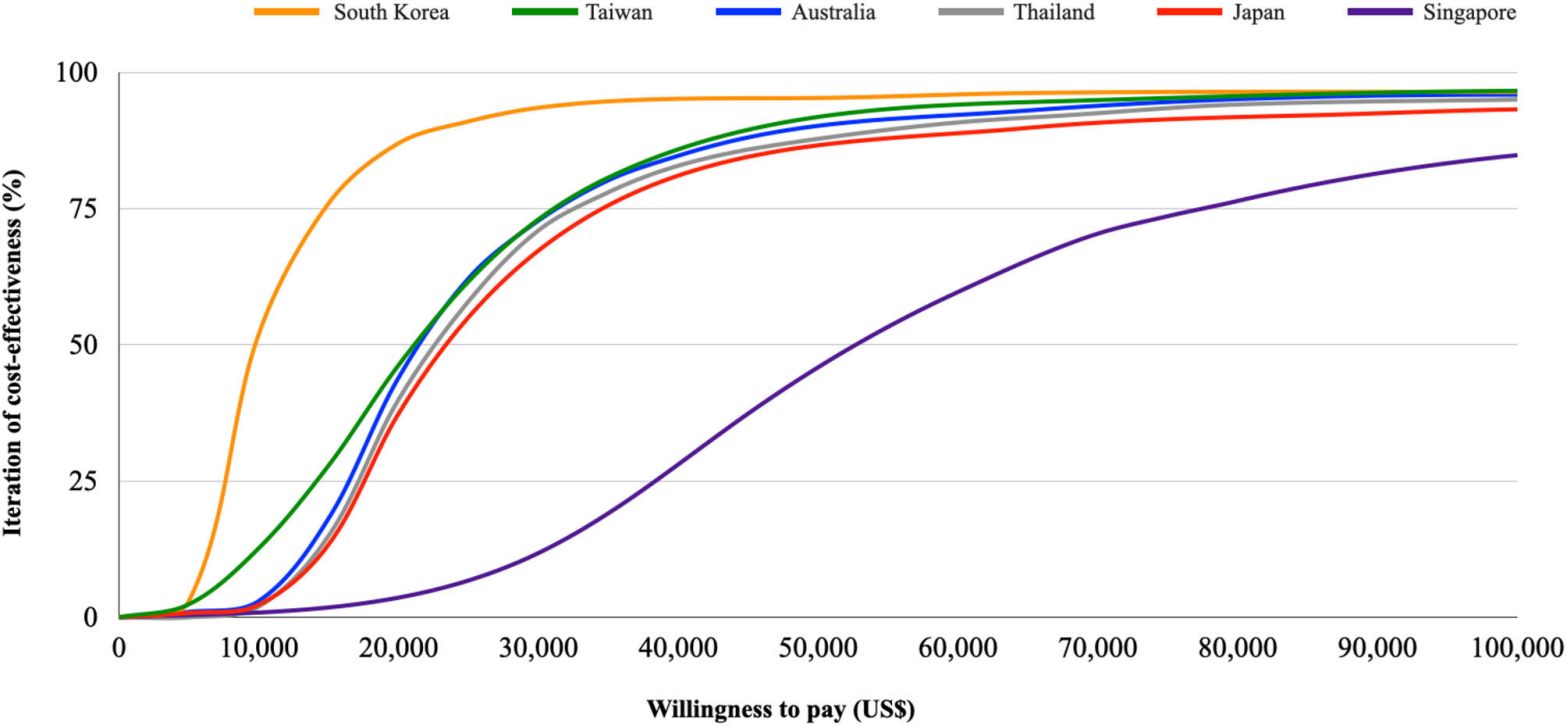
Cost-effectiveness acceptability curve. Iterations of the cost-effectiveness of empagliflozin vs. placebo under different willingness to pay thresholds in Taiwan, Japan, South Korea, Singapore, Thailand, and Australia.

Since the results of cost-effectiveness evaluation are likely to be country- or ethnicity-specific, we reiterated the analyses under the settings of other countries with universal healthcare coverage in the Asia-Pacific region, including Japan, South Korea, Singapore, Thailand, and Australia, to evaluate the cost-effectiveness in the individual countries. We modeled the different costs and utilities from the included countries to compare the pharmacoeconomic benefits from the healthcare system's perspective. The values of the input parameters were extracted from the published sources (Supplementary Table 2) (25–29). Base-case analysis and PSA were both performed for each country. WTP thresholds with 1x and 3x GDP per capita of each country were also used to determine if add-on empagliflozin is a very cost-effective or only a cost-effective option, respectively (21). The cost-effectiveness results are presented in **Table 3**, **Figure 3**.

### Scenario Analyses in Consideration of Adverse Events and Other Variables

Moreover, we constructed the Model 2 (Supplementary Figure 2) to account for the impact of adverse events on our results. The state of hospitalization not only included worsened HF, but also adverse events including hypoglycemia, urinal tract infection, genital infection, bone fracture, and amputation (9). The input variable values are reported in Table 1, Supplementary Tables 1, 2. The cost-effectiveness results are presented in Table 2, and the depicted cost-effectiveness acceptance curve compared to the original model is shown in Supplementary Figure 5.

**Table 2:** The results of base-case cost-effectiveness analysis, probabilistic sensitivity analysis, scenario sensitivity analyses, and subgroup analyses in Taiwan setting.

**In Taiwan setting**	**Costs (US$)**			**QALYs**			**ICERs**	**Result from PSA**	
	**Empagliflozin**	**Placebo**	**Incremental costs**	**Empagliflozin**	**Placebo**	**Incremental QALYs**		**Iteration of WTP threshold at US$ 25,000**	**Iteration of WTP threshold at US$ 75,000**
Base-case analysis	79,141	71,739	7,402	9.66	9.30	0.36	20,508	63.4%	93.7%
Per LY gained instead of QALY gained	79,141	71,739	7,402	12.89	12.42	0.47	15,693	77.9%	95.8%
**Scenario sensitivity analysis**
Account for adverse events	79,542	71,987	7,555	9.56	9.23	0.33	22,581	55.4%	91.1%
Time horizon (30 years)	89,576	80,189	9,387	10.93	10.40	0.54	17,492	77.4%	98.6%
Time horizon (16 months)	15,184	14,232	952	1.86	1.85	0.01	91,617	36.8%	47.1%
Discounting rate at 0%	92,829	83,839	8,990	11.33	10.87	0.46	19,469	68.2%	95.8%
Discounting rate at 10%	57,477	52,479	4,997	7.02	6.80	0.21	23,305	53.8%	90.8%
Risk of cardiovascular mortality of empagliflozin equal to placebo	76,671	71,717	4,954	9.34	9.30	0.04	112,186	20%	45.3%
Risk of non-cardiovascular death for empagliflozin equal to placebo	78,822	71,717	7,105	9.62	9.30	0.32	22,039	57.9%	91.2%
Risk of HHF for empagliflozin equal to placebo	79,385	71,717	7,667	9.66	930	0.36	21,412	59.8%	94.2%
Half the monthly costs of empagliflozin (US$17.5)	76,401	71,717	4,684	9.66	9.30	0.36	12,976	83.6%	96.5%
Half the costs of treatment of hospitalization for heart failure	78,133	70,359	7,774	9.66	9.30	0.36	21,532	59%	93.5%
**Different drug prices of empagliflozin at different WTP thresholds to meet the cost-effectiveness in Taiwan setting**									
**WTP (US$)**	**10,000**	**12,500**	**15,000**	**17,500**	**20,000**		
Monthly costs (US$)	8.2	14	19.5	25.6	31.5		

*QALY, quality-adjusted life year; ICER, incremental cost-effectiveness ratio; PSA, probabilistic sensitivity analyses; WTP, willingness to pay; LY, life years; HHF, hospitalization for heart failure*.

Other scenario sensitivity analyses were performed to account for more considerable influences on ICER among the variables, i.e., time horizon, discount rate, risk of cardiovascular and non-cardiovascular death, risk of HHF, and the costs of drugs and HHF. Half costs of empagliflozin and HHF US$17.5 and US$ 1,443.5 were tested. To provide more information, we also calculated the costs of empagliflozin so that the regimen would be cost-effective at the different WTP thresholds of US$20,000, US$17,500, US$15,000, US$12,500, and US$10,000. Discount rates of 0 and 10% were input to assess the different economic conditions. We used the time horizons of 30 years and 16 months (the follow-up duration in the EMPEROR-Reduced trial) to assess the pharmacoeconomic incentive in the different periods.

### Subgroup Analyses

We further performed subgroup cost-effectiveness analyses according to the EMPEROR-Reduced trial, i.e., aged ≥ 65 or <65 years, varied ethnicities Black, Asian, White), different renal functions (estimated glomerular filtration rate, eGFR ≥ 60 or <60 ml/min/1.73 m^2^), with or without diabetes, ischemic cause for HF, and concomitant use with sacubitril/valsartan (9).

## Results

At the end of the 15-year simulation, the mortality rates were 79.3% in the empagliflozin group and 81.4% in the standard therapy group. For every 1,000 patients with HFrEF treated with empagliflozin, ~296 HHF (803 vs. 1,099) were averted over the 15-year horizon.

Table 2 shows the results of the base-case analysis in which 10 mg of empagliflozin once daily added to standard therapy in patients with HFrEF produced better effectiveness than standard therapy alone (9.66 vs. 9.30 QALYs, and 12.89 vs. 12.42 life years) in the model. Simultaneously, add-on empagliflozin spent more lifetime medical costs (US$79,141 vs. US$71,739). The ICERs in the model were US$20,508 per QALY gained, and US$15,693 per life year gained.

Figure 2 shows the tornado diagram presenting the impact of the different ranges of variables on the ICERs. The probability of cardiovascular death influenced ICER the most, followed by the probability of non-cardiovascular death, monthly costs and utility of stable HF. ICER values were also sensitive to the drug price of empagliflozin, and the probability and costs of HHF.

PSA results are shown in Table 2, Supplementary Figures 3, 4. The likelihood iteration of cost-effectiveness for the empagliflozin regimen vs. standard therapy alone was 63.4 vs. 36.6% at US$25,000, and 93.7 vs. 6.3% at US$75,000 in the Taiwan setting.

Table 2 demonstrates the results of the scenario analyses. With 30 years and 16 months as the time horizon, the ICER became US$17,492 and US$91,617. At discount rates of 0 and 10%, the ICER changed to US$19,469 and US$23,305, respectively. Since cardiovascular death had the strongest influence in the model, we performed a scenario test by assuming the same risk of cardiovascular death in both regimens. Then, the ICER soared up to US$112,186. However, assuming equivalent values of risk for non-cardiovascular death and HHF, the ICER did not change noticeably (US$ 22,039 and US$21,412). At the half costs of empagliflozin and HHF, the ICER changed to US$12,976 and US$21,532. Notably, if the monthly cost of empagliflozin became < US$31.5, US$25.6, US$19.5, US$14.0, and US$8.2, the corresponding ICERs decrease to below the WTP thresholds at US$20,000, US$17,500, US$15,000, US$12,500, and US$10,000. Taking into account the scenario with adverse events, the Model 2 showed that both therapies produced more medical costs and fewer QALYs, and the ICER value increased from US$20,508 to US$22,581, compared to the original model (Supplementary Figure 5).

Table 3, Figure 3 show the results of base-case analysis and PSA for add-on empagliflozin in HFrEF treatment in different Asia-Pacific countries. The Singapore setting resulted in the most costs from the regimen (US$148,751), while the South Korean setting produced the least (US$15,934). The QALYs gained between both therapies did not show much difference across the countries, but all had higher QALYs gained with add-on empagliflozin. The ICER value in the Singapore setting was the highest at US$53,791, followed by Japan (US$24,046), Thailand (US$21,543), Australia (US$20,982), Taiwan (US$20,508), and South Korea (US$8,846). Given the WTP thresholds at 1x and 3x GDP per capita in each country, the likelihood iterations of being a very cost-effective and only a cost-effective option for add-on empagliflozin vs. standard therapy alone were 63.4 and 93.7% for Taiwan, 77.9 and 95.6% for Japan, 93.6 and 96.3% for South Korea, 58.1 and 94.2% for Singapore, 0 and 51.9% for Thailand, and 89 and 95.9% for Australia.

**Table 3:** Base-case analysis and probabilistic sensitivity analysis of cost-effectiveness for add-on empagliflozin vs. standard therapy alone among patients with heart failure and a reduced ejection fraction in the included Asia-Pacific countries.

	**Costs (US$)**			**QALYs**			**ICERs**	**Result from PSA**	
**Asia-Pacific countries**	**Empagliflozin**	**Placebo**	**Incremental costs**	**Empagliflozin**	**Placebo**	**Incremental QALYs**		Iteration of WTP threshold at one-time GDP per capita*	Iteration of WTP threshold at three-times GDP per capita*
Taiwan setting	79,141	71,739	7,402	9.66	9.30	0.36	20,508	63.4%	93.7%
Japan setting	45,210	37,664	7,546	8.37	8.06	0.31	24,046	77.9%	95.6%
South Korea setting	15,934	13,158	2,776	8.37	8.06	0.31	8,846	93.6%	96.3%
Singapore setting	148,751	130,602	188,149	9.02	8.68	0.34	53,791	58.1%	94.2%
Thailand setting	21,805	15,247	6,558	8.11	7.81	0.30	21,543	0%	51.9%
Australia setting	56,356	49,573	6,783	8.63	8.31	0.32	20,982	89%	95.9%

*QALY, quality-adjusted life year; ICER, incremental cost-effectiveness ratio; PSA, probabilistic sensitivity analyses; WTP, willingness to pay; GDP, gross domestic product*.

* *GDP per capita (2020): US$25,000 for Taiwan; US$39,000 for Japan; US$30,000 for South Korea; US$58,000 for Singapore; US$7,300 for Thailand; US$52,000 for Australia*.

In the subgroup analyses, at a WTP threshold of US$25,000, the probabilities of cost-effectiveness of add-on empagliflozin therapy were similar irrespective of the subjects being older or younger than 65 years. The regimen had the highest probability of being cost-effective for Black people, followed by Asians and White people. In the subpopulation with diabetes, eGFR ≥ 60 ml/min/1.73m^2^, non-ischemic HF, NYHA Fc II, or concomitant sacubitril/valsartan use, add-on empagliflozin therapy was more cost-effective at the WTP threshold of US$25,000, compared to those without diabetes or sacubitril/valsartan use and those with eGFR <60 ml/min/1.73 m^2^, ischemic HF, or NYHA Fc III-IV (Supplementary Table 4, Supplementary Figure 6).

## Discussion

From our results, the incremental costs per QALY and life year gained in the base-case analysis and PSA were lower than 1x and 3x the GDP per capita in Taiwan, Japan, Singapore, South Korea, and Australia. Namely, adding empagliflozin to standard therapy was likely to be a very cost-effective add-on therapy from a national payer's perspective in these countries. Regarding Thailand, ICER is only lower than 3x the GDP per capita, and thus, adding empagliflozin is only a cost-effective option. Although the incremental cost per life year gained was lower than the incremental cost per QALY gained, using this ICER value may lead to an underestimation. Mainly, chronic clinical events severely influence the quality of life. For example, the utility of heart failure is only 0.77 of one perfectly healthy year. In comparison to life years, QALY provides a more appropriate evaluation of the cost-effectiveness of new treatments for chronic diseases (30).

In the one-way sensitivity analysis, the risk of cardiovascular death had the most significant impact on the ICERs. To assess the impact of the parameters, we used the hypothetical scenario to evaluate the ICERs. In the EMPEROR-Reduced trial, the add-on empagliflozin regimen had a lower risk of cardiovascular death than in the placebo group (hazard ratio 0.92, 95% confidence interval 0.75–1.12), despite the difference being statistically insignificant (9). If the cardiovascular death risk was the same in both therapies, the add-on empagliflozin regimen would not have pharmacoeconomic incentives (ICER of US$112,186), meaning that the pharmacoeconomic benefits may need to be carefully re-assessed with real-world data after the initial use of empagliflozin in HFrEF patients. However, given the same risks of non-cardiovascular death and HHF for both therapies, empagliflozin remained a very cost-effective therapy in HFrEF treatment. On the other hand, costs of empagliflozin substantially influenced the pharmacoeconomic benefits, while HHF costs had less influence. For example, at the half-cost of empagliflozin and HHF, ICER values became US$12,976 and US$21,532 per QALY gained, respectively. This hypothetic scenario may provide reference to support that negotiating the drug price may result in more pharmacoeconomic benefits than adjusting the healthcare costs for HHF. In addition, the current study also estimated the appropriate drug price to meet the cost-effectiveness of empagliflozin given the different WTP thresholds from US$10,000 to US$25,000. The results also provide scientific references for policymaking or bargaining drug costs for the target population.

In another scenario analysis, ICER values remained < US$25,000 regardless of the different discount rates (0–10%), which may strengthen the robustness of the cost-effectiveness of empagliflozin when accounting for the time factor. Different time horizons of 30 years and 16 months were applied to take into consideration the super-aged society and the follow-up period of the EMPEROR-Reduced trial. We observed that the longer the time horizon, the smaller the ICER values, indicating that the regimen has more pharmacoeconomic incentives in long-term use. In addition, we constructed the Model 2 to simulate the complexity accounting for the impact of adverse events. Although the ICER increased from US$20,508 to US$22,581, the pharmacoeconomic benefit was still attractive. This may be consistent with the condition that except for uncomplicated genital tract infections, most adverse events did not show significant differences between the two therapies (9). In the subgroup analysis, we found that using add-on empagliflozin in the Black and Asian population with HFrEF was likely to yield more pharmacoeconomic benefits, compared to Caucasians. Furthermore, the populations with diabetes, eGFR ≥ 60 ml/min/1.73 m^2^, non-ischemic HF, NYHA Fc II, or concomitant sacubitril/valsartan use were likely to show more benefits in terms of cost-effectiveness. Policymakers may prioritize specific patient groups for the add-on therapy in HFrEF under the financial constraints of the healthcare system (Supplementary Table 4, Supplementary Figure 6).

In our study, the pharmacoeconomic attraction differs across the different country settings. South Korea had the lowest ICER value (US$8,846) mainly due to the lowest drug cost of empagliflozin. Conversely, Singapore had the highest ICER value (US$53,791), which may be due to the expensive medical spending. However, despite the higher medical expenditure, empagliflozin is still highly possible to be a very cost-effective treatment in the Singapore setting due to the higher WTP threshold. Likewise, although medical costs were lower in Thailand with a median ICER value in the Asia-Pacific region, the regimen would be only cost-effective due to the lower WTP threshold.

To the best of our knowledge, the current analysis is the first to assess the cost-effectiveness of empagliflozin added to standard therapy in patients with HFrEF. Dapagliflozin has been shown to be cost-effective add-on therapy for patients with HFrEF in the U.K. (ICER £5822/QALY gained), Germany (ICER €5379/QALY gained), Spain (ICER €9406/QALY gained), and Australia (ICER A$12,482/QALY gained) (10, 11) Comparing the two SGLT2 inhibitors in HFrEF treatment, the ICER yielded by empagliflozin was likely to be greater than that yielded by dapagliflozin in spite of the different settings, e.g., A$12,482/QALY gained for dapagliflozin vs. US$20,982/QALY gained for empagliflozin in the Australia setting. The disparity in the clinical efficacy of cardiovascular and non-cardiovascular death might be the major influence (31).

There are some limitations of the current study. First, the study parameters were collected from several sources, which may contribute to the uncertainty. However, we derived the clinical transitional probabilities from only the EMPEROR-Reduced trial. The design of double-blinded randomized controlled trial may help to mitigate the uncertainty and provide convincing evidence (32). Besides, we tested all input parameters in various sensitivity analyses, and the pharmacoeconomic conclusion did not change. Second, using parameters from different races might lead to uncertainty because Asians composed only 13–14% of all subjects in the trial. Nevertheless, the PSA using Monte Carlo Model considered a different and wide range of the transitional probabilities, which may cover the probabilities in different races. Besides, the hazard ratio of primary outcomes for Asians was 0.57 (0.41–0.78), which was better than the entire enrolled population [0.75, (0.65–0.68)] (14). If we only used the variables from Asian populations, the pharmacoeconomic benefit would become more positive in our analyses (Supplementary Table 4, Supplementary Figure 6). Third, Taiwan and some Asia-Pacific countries have not reached a public consensus on the WTP threshold, and the pharmacoeconomic incentives may differ according to the different thresholds. Thus, the study provided the iteration of the cost-effectiveness using different WTP thresholds in Asia-Pacific countries (from US$0 to US$100,000) to ameliorate the concern (Figure 3). Fourth, the model might simplify the real-world conditions. For example, the current study assumed that the influence of adverse events was neglected, and the transitional probabilities were constant irrespective of the comorbidities, recurrent diseases, and aging. The assumption may not be sufficient to reflect the possible changes in the risks of disease progression or death over time with the aging of patients in a chronic disease course of HF. To ameliorate the concerns, we performed a series of sensitivity analyses, including PSA with a varied range of transitional probabilities and scenario analyses with different time horizons. Besides, we took account of the adverse events in the Model 2 for base-case analysis and PSA. In these analyses, the positive conclusions did not change. The abovementioned consistent findings between these base-case analyses and further analyses strengthened the robustness of the pharmacoeconomic benefits.

Finally, the current analysis was performed from the perspective of a national healthcare system, and the costs only included direct medical costs. Although the different level of cost resources, treatment context, and WTP thresholds across different countries would influence the cost-effectiveness results (national-level medical costs database in Taiwan, South Korea, Singapore, Australia, and hospital-level data in Japan and Thailand) (22–26), the current study extracted the data all from the healthcare system's perspective and further performed PSA with gamma distribution covering a wide and varied range of costs to strengthen the robustness of findings. Also, the study provided the different probabilities of cost-effectiveness for add-on empagliflozin vs. standard care alone in HFrEF treatment under various WTP thresholds. Nevertheless, the health technique assessment of add-on empagliflozin in HFrEF treatment may still require a more comprehensive evaluation by considering the financial strains of the healthcare system, reimbursement policy, societal costs, opportunity costs, equity, and equality. Further studies are also needed to consider the costs from a societal perspective, such as indirect medical costs, productivity loss, and social services.

## Conclusions

In conclusion, our results showed that add-on empagliflozin in patients with HFrEF produced improved effectiveness accompanied with acceptable costs. Although empagliflozin is likely to be a cost-effective treatment for HFrEF, the pharmacoeconomic benefits are influenced by the WTP thresholds across different healthcare systems in the Asia-Pacific region.

## Data Availability Statement

Publicly available datasets were analyzed in this study. This data can be found at: Taiwan National Health Insurance Database.

## Author Contributions

C-TL and C-TY contributed to this study, including the conception and design of the work, and drafting of the manuscript. F-HK, M-CL, and W-TC contributed to the acquisition, analysis, and interpretation of data for this work. H-TO, H-JT, and Y-MH contributed to modifying the model structure. Z-CC, H-YC, CS, and HST critically revised the manuscript. All authors gave final approval and agreed to be accountable for all aspects of work ensuring integrity and accuracy.

## Conflict of Interest

The authors declare that the research was conducted in the absence of any commercial or financial relationships that could be construed as a potential conflict of interest.

## Publisher's Note

All claims expressed in this article are solely those of the authors and do not necessarily represent those of their affiliated organizations, or those of the publisher, the editors and the reviewers. Any product that may be evaluated in this article, or claim that may be made by its manufacturer, is not guaranteed or endorsed by the publisher.
