# Supplementary material for: Cost-Effectiveness Evaluation of Add-on Empagliflozin in Patients With Heart Failure and a Reduced Ejection Fraction From the Healthcare System's Perspective in the Asia-Pacific Region
Source: Front Cardiovasc Med. 2021 Oct 29;8:750381. doi: 10.3389/fcvm.2021.750381 (PMC8586201; doi:10.3389/fcvm.2021.750381)
Supplement: Supplementary file 1 [file Table_1.docx]

| **Supplementary Table 1.**  **Monthly transitional probability of adverse events for empagliflozin added to standard therapy and standard therapy alone in Model 2** | | | | |
| --- | --- | --- | --- | --- |
|  | **Add-on empagliflozin**  **(Monthly transitional probability ± standard error)** | **Standard therapy alone**  **(Monthly transitional probability ± standard error)** | **Distribution** | **Reference** |
| **Adverse Events** | | | | |
| **Hypoglycemia** | 0.000912009 ± 0.000699351 | 0.000943987 ± 0.000710732 | Beta | EMPEROR-Reduced |
| **Urinary tract infection** | 0.003125059 ± 0.001293133 | 0.002838142 ± 0.001231197 | Beta | EMPEROR-Reduced |
| **Genital infection** | 0.001048189 ± 0.000749697 | 0.000402929 ± 0.000464467 | Beta | EMPEROR-Reduced |
| **Bone fracture** | 0.001527026 ± 0.000904659 | 0.001421044 ± 0.000871812 | Beta | EMPEROR-Reduced |
| **Amputation** | 0.000437558 ± 0.000484525 | 0.000335605 ± 0.000423906 | Beta | EMPEROR-Reduced |
| The transitional parameters were extracted from the EMPEROR-Reduced trial.  The monthly transition probabilities were transformed by the following process:  (1) Probability (obtained from the EMPEROR-Reduced trial) to a rate = [-ln (1-p)] ÷ t  (2) Rate to a probability (monthly transition probability applied in the analyses) = 1- exp(-rt)  Where r = rate, p = probability, and t = time. | | | | |
