# Supplementary material for: Cost-Effectiveness Evaluation of Add-on Empagliflozin in Patients With Heart Failure and a Reduced Ejection Fraction From the Healthcare System's Perspective in the Asia-Pacific Region
Source: Front Cardiovasc Med. 2021 Oct 29;8:750381. doi: 10.3389/fcvm.2021.750381 (PMC8586201; doi:10.3389/fcvm.2021.750381)
Supplement: Supplementary file 2 [file Table_2.docx]

| **Supplementary Table 2.**  **Input parameters of utilities and costs for scenario analysis in the Model 2 in Taiwan setting** | | | | |
| --- | --- | --- | --- | --- |
| **Variables** | **Estimates** | **Standard error** | **Distribution** | **Reference** |
| **Utility score** | | | | |
| Hypoglycemia | -0.014 | 0.001 | Beta | [1], [2] |
| Urinary tract infection | -0.003 | 0.001 | Beta |  |
| Genital infection | -0.003 | 0.001 | Beta |  |
| Bone fracture | -0.148 | 0.033 | Beta |  |
| Amputation | -0.28 | 0.053 | Beta |  |
| **Monthly Costs (US$)** | **Costs (US$)** | **Range** | **Distribution** |  |
| Urinary tract infection | 560 | 280 | Gamma | NHIRD |
| Genital infection | 560 | 280 | Gamma |  |
| Bone fracture | 1,350 | 675 | Gamma |  |
| Amputation | 7,877 | 3,938.5 | Gamma |  |
| NHIRD, National Health Insurance Research Database.  1. Sullivan PW, et. al Medical Decision Making. 2006;26(4):410-420.  2. Lee MC, et al. Cardiovascular drugs and therapy. 2020:1-9. | | | | |
|  | | | | |
