# Supplementary material for: Cost-Effectiveness Evaluation of Add-on Empagliflozin in Patients With Heart Failure and a Reduced Ejection Fraction From the Healthcare System's Perspective in the Asia-Pacific Region
Source: Front Cardiovasc Med. 2021 Oct 29;8:750381. doi: 10.3389/fcvm.2021.750381 (PMC8586201; doi:10.3389/fcvm.2021.750381)
Supplement: Supplementary file 3 [file Table_3.docx]

| **Supplementary Table 3.**  **Type of healthcare system, utility of heart failure and relevant cost references in different Asia-Pacific countries** | | | | | |  |
| --- | --- | --- | --- | --- | --- | --- |
|  | **Taiwan** | **Japan^1^** | **South Korea^2^** | **Singapore^3^** | **Thailand^4^** | **Australia^5^** |
| **Healthcare system** | Universal  healthcare coverage | Universal  healthcare coverage | Universal  healthcare coverage | Universal  healthcare coverage | Universal  healthcare coverage | Universal  healthcare coverage |
| **Utility score of heart failure** | 0.77 | 0.67 | 0.67 | 0.72 | 0.75 | 0.69 |
| **Monthly costs of empagliflozin (US$)** | 35 | 63 | 18.2 | 90 | 39 | 46.8 |
| **Monthly costs for stable heart failure (US$)** | 450 | 115 | 70 | 835 | 98 | 267 |
| **Costs of hospitalization for heart failure (US$)** | 2,887 | 10,234 | 1,217 | 3,403 | 370 | 8,048 |
| **Costs one month before death (US$)** | 3,430 | 15,804 | 1,824 | 5,378 | 555 | 3,538 |
| **Cost resources (whose perspective)** | National database (Healthcare payer’s perspective) | Hospital-level database (Healthcare payer’s perspective) | National database (Healthcare payer’s perspective) | National database (Healthcare payer’s perspective) | Hospital-level database (Healthcare payer’s perspective) | National database (Healthcare payer’s perspective) |
| ^1^ Circulation Journal. 2004;68(1):35-40.  ^2^ Clinical therapeutics. 2019;41(6):1066-1079.  ^3^ Journal of medical economics. 2018;21(2):174-181  ^4^ Clinicoeconomics and Outcomes Research: CEOR. 2020; 12:579.  ^5^ Heart, Lung and Circulation. 2020;29(9):1310-1317. | | | | | | |
