# Supplementary material for: Cost-Effectiveness Evaluation of Add-on Empagliflozin in Patients With Heart Failure and a Reduced Ejection Fraction From the Healthcare System's Perspective in the Asia-Pacific Region
Source: Front Cardiovasc Med. 2021 Oct 29;8:750381. doi: 10.3389/fcvm.2021.750381 (PMC8586201; doi:10.3389/fcvm.2021.750381)
Supplement: Supplementary file 4 [file Table_4.docx]

| **Supplementary Table 4.**  **Iteration of cost-effectiveness analyses of empagliflozin versus placebo stratified by age, ethnicity, eGFR, diabetes, cause of heart failure, NYHA functional class, use of sacubitril/valsartan** | | | | | | | | | | | | | | | | |
| --- | --- | --- | --- | --- | --- | --- | --- | --- | --- | --- | --- | --- | --- | --- | --- | --- |
|  |  | **Age** | | **Ethnicity** | | | **eGFR** | | **Diabetes** | | **Ischemic** | | **NYHA functional class** | | **sacubitril/valsartan use** | |
| **WTP** | **Strategy** | <65 yr | ≥65yr | Black | Asia | White | ≥60 | <60 | Yes | No | Yes | No | II | III or IV | Yes | No |
| 0 | EMPA | 0 | 0 | 0 | 0 | 0.008 | 0 | 0.003 | 0 | 0.001 | 0.004 | 0 | 0 | 0.003 | 0 | 0.001 |
| 0 | placebo | 1 | 1 | 1 | 1 | 0.992 | 1 | 0.997 | 1 | 0.999 | 0.996 | 1 | 1 | 0.997 | 1 | 0.999 |
| 5,000 | EMPA | 0.007 | 0.007 | 0 | 0 | 0.03 | 0.004 | 0.017 | 0.006 | 0.018 | 0.022 | 0.005 | 0.007 | 0.02 | 0.001 | 0.009 |
| 5,000 | placebo | 0.993 | 0.993 | 1 | 1 | 0.97 | 0.996 | 0.983 | 0.994 | 0.982 | 0.978 | 0.995 | 0.993 | 0.98 | 0.999 | 0.991 |
| 10,000 | EMPA | 0.492 | 0.484 | 0.845 | 0.693 | 0.19 | 0.485 | 0.266 | 0.423 | 0.317 | 0.282 | 0.508 | 0.432 | 0.252 | 0.553 | 0.328 |
| 10,000 | placebo | 0.508 | 0.516 | 0.155 | 0.307 | 0.81 | 0.515 | 0.734 | 0.577 | 0.683 | 0.718 | 0.492 | 0.568 | 0.748 | 0.447 | 0.672 |
| 15,000 | EMPA | 0.918 | 0.909 | 1 | 0.996 | 0.489 | 0.981 | 0.661 | 0.947 | 0.824 | 0.731 | 0.983 | 0.953 | 0.668 | 0.98 | 0.866 |
| 15,000 | placebo | 0.082 | 0.091 | 0 | 0.004 | 0.511 | 0.019 | 0.339 | 0.053 | 0.176 | 0.269 | 0.017 | 0.047 | 0.332 | 0.02 | 0.134 |
| 20,000 | EMPA | 0.977 | 0.971 | 1 | 1 | 0.689 | 1 | 0.839 | 0.996 | 0.955 | 0.886 | 0.999 | 0.997 | 0.819 | 0.997 | 0.985 |
| 20,000 | placebo | 0.023 | 0.029 | 0 | 0 | 0.311 | 0 | 0.161 | 0.004 | 0.045 | 0.114 | 0.001 | 0.003 | 0.181 | 0.003 | 0.015 |
| 25,000 | EMPA | 0.995 | 0.988 | 1 | 1 | 0.789 | 1 | 0.907 | 0.999 | 0.984 | 0.941 | 1 | 1 | 0.878 | 0.999 | 0.996 |
| 25,000 | placebo | 0.005 | 0.012 | 0 | 0 | 0.211 | 0 | 0.093 | 0.001 | 0.016 | 0.059 | 0 | 0 | 0.122 | 0.001 | 0.004 |
| 30,000 | EMPA | 0.999 | 0.996 | 1 | 1 | 0.85 | 1 | 0.942 | 1 | 0.993 | 0.966 | 1 | 1 | 0.919 | 0.999 | 0.997 |
| 30,000 | placebo | 0.001 | 0.004 | 0 | 0 | 0.15 | 0 | 0.058 | 0 | 0.007 | 0.034 | 0 | 0 | 0.081 | 0.001 | 0.003 |
| 35,000 | EMPA | 1 | 0.998 | 1 | 1 | 0.875 | 1 | 0.953 | 1 | 0.994 | 0.979 | 1 | 1 | 0.941 | 1 | 0.998 |
| 35,000 | placebo | 0 | 0.002 | 0 | 0 | 0.125 | 0 | 0.047 | 0 | 0.006 | 0.021 | 0 | 0 | 0.059 | 0 | 0.002 |
| 40,000 | EMPA | 1 | 0.998 | 1 | 1 | 0.897 | 1 | 0.966 | 1 | 0.997 | 0.983 | 1 | 1 | 0.953 | 1 | 1 |
| 40,000 | placebo | 0 | 0.002 | 0 | 0 | 0.103 | 0 | 0.034 | 0 | 0.003 | 0.017 | 0 | 0 | 0.047 | 0 | 0 |
| 45,000 | EMPA | 1 | 0.999 | 1 | 1 | 0.911 | 1 | 0.971 | 1 | 0.999 | 0.989 | 1 | 1 | 0.959 | 1 | 1 |
| 45,000 | placebo | 0 | 0.001 | 0 | 0 | 0.089 | 0 | 0.029 | 0 | 0.001 | 0.011 | 0 | 0 | 0.041 | 0 | 0 |
| 50,000 | EMPA | 1 | 0.999 | 1 | 1 | 0.92 | 1 | 0.977 | 1 | 0.999 | 0.991 | 1 | 1 | 0.963 | 1 | 1 |
| 50,000 | placebo | 0 | 0.001 | 0 | 0 | 0.08 | 0 | 0.023 | 0 | 0.001 | 0.009 | 0 | 0 | 0.037 | 0 | 0 |
| 55,000 | EMPA | 1 | 0.999 | 1 | 1 | 0.929 | 1 | 0.98 | 1 | 0.999 | 0.993 | 1 | 1 | 0.969 | 1 | 1 |
| 55,000 | placebo | 0 | 0.001 | 0 | 0 | 0.071 | 0 | 0.02 | 0 | 0.001 | 0.007 | 0 | 0 | 0.031 | 0 | 0 |
| 60,000 | EMPA | 1 | 0.999 | 1 | 1 | 0.937 | 1 | 0.981 | 1 | 0.999 | 0.994 | 1 | 1 | 0.97 | 1 | 1 |
| 60,000 | placebo | 0 | 0.001 | 0 | 0 | 0.063 | 0 | 0.019 | 0 | 0.001 | 0.006 | 0 | 0 | 0.03 | 0 | 0 |
| 65,000 | EMPA | 1 | 0.999 | 1 | 1 | 0.942 | 1 | 0.983 | 1 | 0.999 | 0.994 | 1 | 1 | 0.974 | 1 | 1 |
| 65,000 | placebo | 0 | 0.001 | 0 | 0 | 0.058 | 0 | 0.017 | 0 | 0.001 | 0.006 | 0 | 0 | 0.026 | 0 | 0 |
| 70,000 | EMPA | 1 | 1 | 1 | 1 | 0.945 | 1 | 0.985 | 1 | 0.999 | 0.994 | 1 | 1 | 0.976 | 1 | 1 |
| 70,000 | placebo | 0 | 0 | 0 | 0 | 0.055 | 0 | 0.015 | 0 | 0.001 | 0.006 | 0 | 0 | 0.024 | 0 | 0 |
| 75,000 | EMPA | 1 | 1 | 1 | 1 | 0.949 | 1 | 0.985 | 1 | 0.999 | 0.994 | 1 | 1 | 0.977 | 1 | 1 |
| 75,000 | placebo | 0 | 0 | 0 | 0 | 0.051 | 0 | 0.015 | 0 | 0.001 | 0.006 | 0 | 0 | 0.023 | 0 | 0 |
| 80,000 | EMPA | 1 | 1 | 1 | 1 | 0.952 | 1 | 0.985 | 1 | 0.999 | 0.994 | 1 | 1 | 0.978 | 1 | 1 |
| 80,000 | placebo | 0 | 0 | 0 | 0 | 0.048 | 0 | 0.015 | 0 | 0.001 | 0.006 | 0 | 0 | 0.022 | 0 | 0 |
| 85,000 | EMPA | 1 | 1 | 1 | 1 | 0.953 | 1 | 0.985 | 1 | 0.999 | 0.994 | 1 | 1 | 0.979 | 1 | 1 |
| 85,000 | placebo | 0 | 0 | 0 | 0 | 0.047 | 0 | 0.015 | 0 | 0.001 | 0.006 | 0 | 0 | 0.021 | 0 | 0 |
| 90,000 | EMPA | 1 | 1 | 1 | 1 | 0.953 | 1 | 0.987 | 1 | 0.999 | 0.995 | 1 | 1 | 0.979 | 1 | 1 |
| 90,000 | placebo | 0 | 0 | 0 | 0 | 0.047 | 0 | 0.013 | 0 | 0.001 | 0.005 | 0 | 0 | 0.021 | 0 | 0 |
| 95,000 | EMPA | 1 | 1 | 1 | 1 | 0.953 | 1 | 0.988 | 1 | 0.999 | 0.995 | 1 | 1 | 0.979 | 1 | 1 |
| 95,000 | placebo | 0 | 0 | 0 | 0 | 0.047 | 0 | 0.012 | 0 | 0.001 | 0.005 | 0 | 0 | 0.021 | 0 | 0 |
| 100,000 | EMPA | 1 | 1 | 1 | 1 | 0.953 | 1 | 0.988 | 1 | 0.999 | 0.995 | 1 | 1 | 0.979 | 1 | 1 |
| 100,000 | placebo | 0 | 0 | 0 | 0 | 0.047 | 0 | 0.012 | 0 | 0.001 | 0.005 | 0 | 0 | 0.021 | 0 | 0 |
| EMPA, empagliflozin; WTP, willingness to pay; eGFR, estimated glomerular filtration rate; NYHA, New York Heart Association. | | | | | | | | | | | | | | | | |
