# Supplementary material for: Cost-Effectiveness Evaluation of Add-on Empagliflozin in Patients With Heart Failure and a Reduced Ejection Fraction From the Healthcare System's Perspective in the Asia-Pacific Region
Source: Front Cardiovasc Med. 2021 Oct 29;8:750381. doi: 10.3389/fcvm.2021.750381 (PMC8586201; doi:10.3389/fcvm.2021.750381)
Supplement: Supplementary file 6 [file Image_2.pdf]

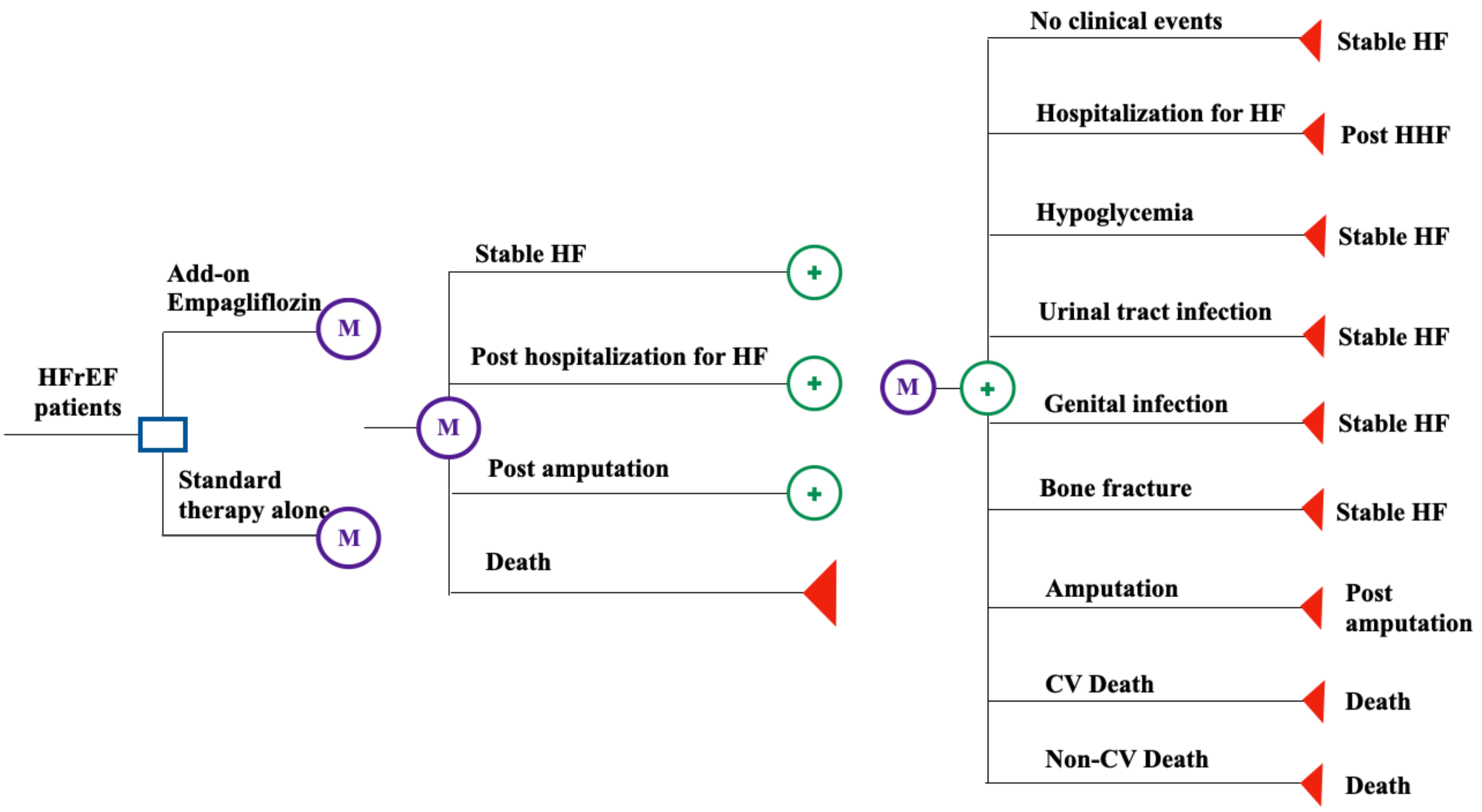

Supplementary Figure 2. Overview of detailed structure of the Model 2 in cost-effectiveness analysis where adverse events of treatment were considered. HFrEF, heart failure and a reduced ejection fraction; HF, heart failure; HHF, hospitalization for heart failure; CV, cardiovascular.
