# Supplementary material for: Cost-Effectiveness Evaluation of Add-on Empagliflozin in Patients With Heart Failure and a Reduced Ejection Fraction From the Healthcare System's Perspective in the Asia-Pacific Region
Source: Front Cardiovasc Med. 2021 Oct 29;8:750381. doi: 10.3389/fcvm.2021.750381 (PMC8586201; doi:10.3389/fcvm.2021.750381)
Supplement: Supplementary file 7 [file Image_3.pdf]

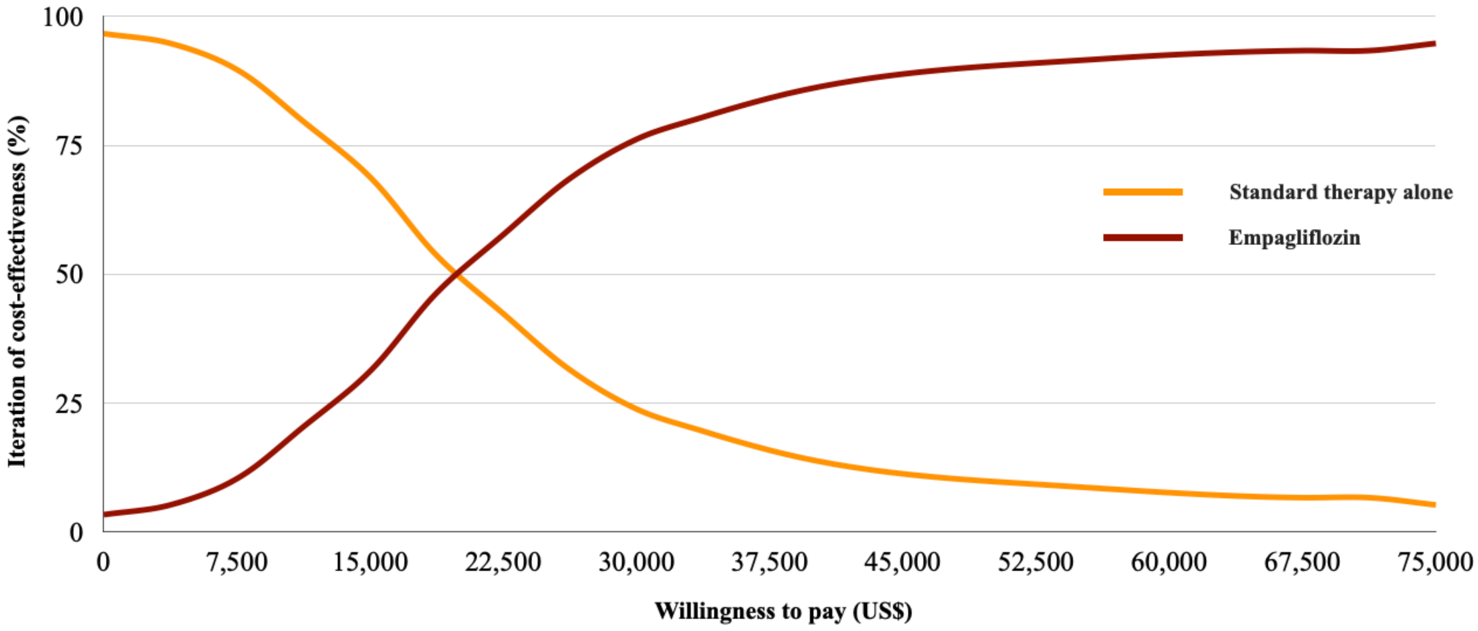

Supplementary Figure 3. Cost-effectiveness acceptability curves. Iterations of the cost-effectiveness of empagliflozin versus placebo under different willingness to pay thresholds in Taiwan. The red curve is for the population with add-on empagliflozin; the orange curve is for the population with standard therapy.
