# Supplementary material for: Cost-Effectiveness Evaluation of Add-on Empagliflozin in Patients With Heart Failure and a Reduced Ejection Fraction From the Healthcare System's Perspective in the Asia-Pacific Region
Source: Front Cardiovasc Med. 2021 Oct 29;8:750381. doi: 10.3389/fcvm.2021.750381 (PMC8586201; doi:10.3389/fcvm.2021.750381)
Supplement: Supplementary file 9 [file Image_5.pdf]

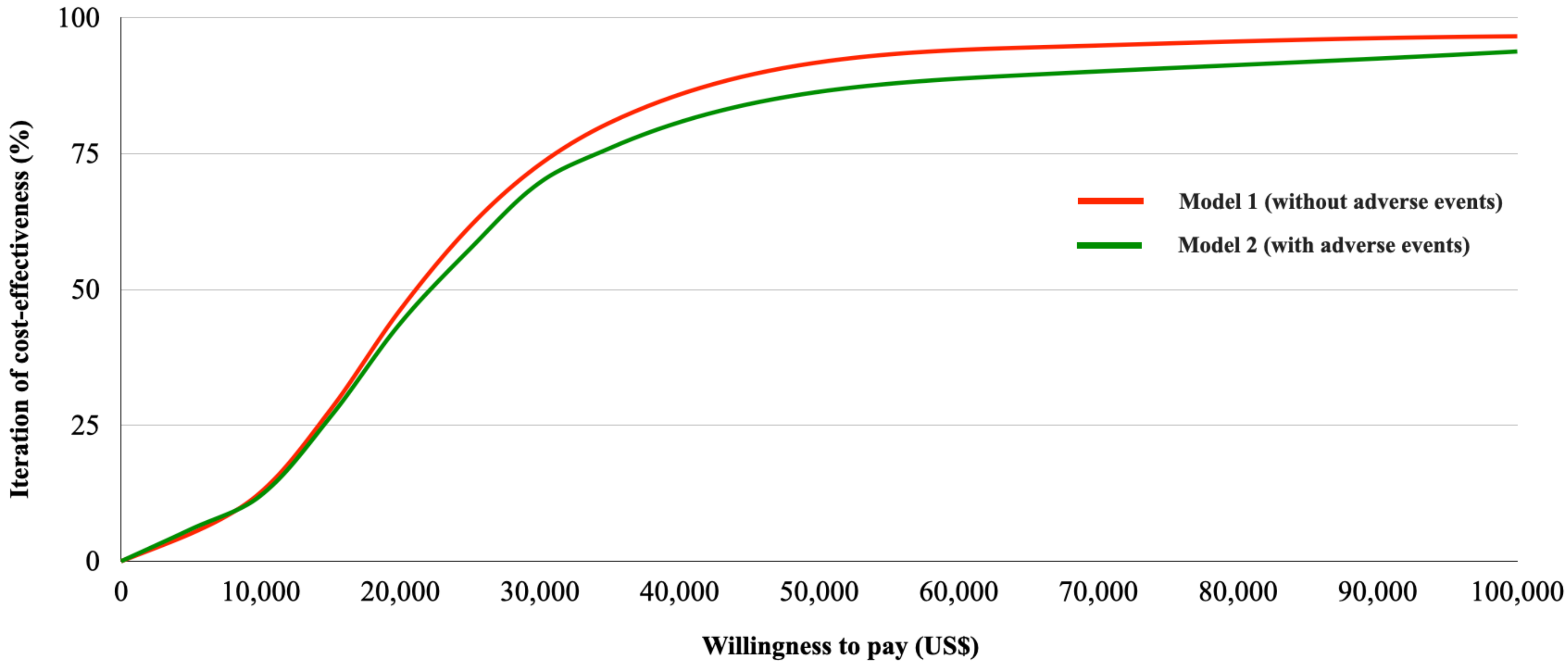

Supplementary Figure 5. Cost-effectiveness acceptability curves. Iterations of the cost-effectiveness of empagliflozin versus placebo under different willingness to pay thresholds in models with and without adverse events. The red curve is the original model (Model 1) without accounting for adverse events; the green curve is the Model 2 accounting for adverse events
