# Supplementary material for: Cost-Effectiveness Evaluation of Add-on Empagliflozin in Patients With Heart Failure and a Reduced Ejection Fraction From the Healthcare System's Perspective in the Asia-Pacific Region
Source: Front Cardiovasc Med. 2021 Oct 29;8:750381. doi: 10.3389/fcvm.2021.750381 (PMC8586201; doi:10.3389/fcvm.2021.750381)
Supplement: Supplementary file 10 [file Image_6.pdf]

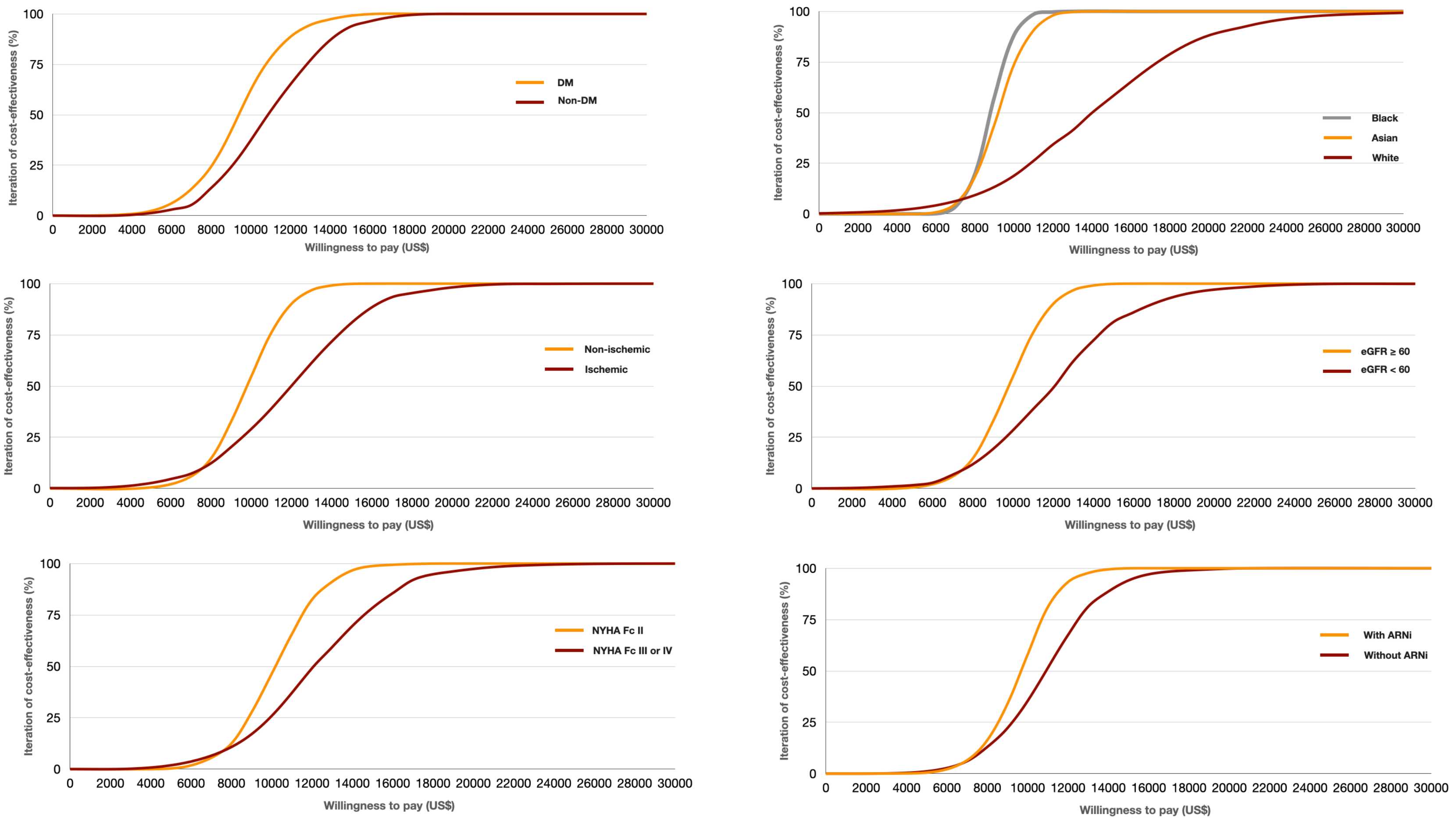

Supplementary Figure 6. Cost-effectiveness acceptability curves. Iterations of the cost-effectiveness of empagliflozin versus placebo in the subgroup analyses.
